# From uncertainty to reward: BOLD characteristics differentiate signaling pathways

**DOI:** 10.1186/1471-2202-10-154

**Published:** 2009-12-22

**Authors:** Birgit Abler, Bärbel Herrnberger, Georg Grön, Manfred Spitzer

**Affiliations:** 1 Department of Psychiatry, University of Ulm, Leimgrubenweg 12-14, 89075 Ulm, Germany

## Abstract

**Background:**

Reward value and uncertainty are represented by dopamine neurons in monkeys by distinct phasic and tonic firing rates. Knowledge about the underlying differential dopaminergic pathways is crucial for a better understanding of dopamine-related processes. Using functional magnetic resonance blood-oxygen level dependent (BOLD) imaging we analyzed brain activation in 15 healthy, male subjects performing a gambling task, upon expectation of potential monetary rewards at different reward values and levels of uncertainty.

**Results:**

Consistent with previous studies, ventral striatal activation was related to both reward magnitudes and values. Activation in medial and lateral orbitofrontal brain areas was best predicted by reward uncertainty. Moreover, late BOLD responses relative to trial onset were due to expectation of different reward values and likely to represent phasic dopaminergic signaling. Early BOLD responses were due to different levels of reward uncertainty and likely to represent tonic dopaminergic signals.

**Conclusions:**

We conclude that differential dopaminergic signaling as revealed in animal studies is not only represented locally by involvement of distinct brain regions but also by distinct BOLD signal characteristics.

## Background

The perceived uncertainty of events is an important parameter modulating decision-making in politics, economy and every-day life. Theories of economics [1,2] defined basic terms to characterize reward and decision making processes: first, the expected value, calculated as the product of magnitude and probability of a certain monetary gain (or loss), and second, the uncertainty of the outcome. Uncertainty in terms of reward processes can be assumed to be maximal when the probability of a reward is 50% and minimal when the probability is either 0% or 100%.

Likewise, recent animal experiments [3] and brain imaging studies of the reward system in human subjects [4-6] suggest two different systems coding reward value and uncertainty based on dopaminergic transmission. In monkeys, Fiorillo et al. [3] demonstrated two different types dopaminergic cell responses coding distinct properties of rewards: a phasic response coding for the expected reward value (probability × magnitude), and a tonic response coding reward uncertainty. In this context, the tonic signal was defined as the difference in firing rates of dopaminergic neurons when comparing trials of higher uncertainty to those with no uncertainty. As uncertainty also correlates with the latency until the reward is delivered, this signal increased gradually with increasing time. Schultz [7,8] suggested that these phasic and tonic responses activate two different types of dopamine receptors: D1 receptors characterized by a low-affinity state to dopamine would be activated by short-lasting, but relatively high peak concentrations of dopamine related to the expected value (phasic response). Conversely, activation of D2 receptors would be correlated by longer-lasting responses related to reward uncertainties (tonic response).

Previous functional magnetic resonance imaging (fMRI) studies have suggested that the expected value of a reward is coded in the ventral striatum [9-11] and medial orbitofrontal regions [12,13]. Reward uncertainty was suggested to be coded in lateral aspects of the orbitofrontal cortex [4,6], but also the insula [14], the medial prefrontal cortex [15] and the prefrontal cortex [16]. Additionally, it has been proposed [4] that the different dopaminergic pathways involved may be reflected in different signaling time courses: early signals in the lateral orbitofrontal and late signals in striatal regions.

Consequently we designed an experiment that allowed us to investigate the coding of reward uncertainty independent of reward value varying degrees of uncertainty systematically over a range of five probabilities. Intending to replicate animal research as close as possible and to determine the suggested differential fMRI signal characteristics of reward uncertainty and value when varied independently in human subjects, we hypothesized that

(1) reward uncertainty related to tonic dopaminergic signals results in relatively early BOLD responses in lateral orbitofrontal regions, while

(2) expected reward value or its components, reward magnitude and probability, related to phasic signals are coded independently of uncertainty in the ventral striatum by a comparably later BOLD response.

## Methods

15 healthy male right-handed subjects (age range: 23-27 years) with no history of psychiatric or neurological disease gave written informed consent. The study was approved by the ethics committee of the University of Ulm. Before scanning, all subjects completed a practice version of the task.

### Task

**Figure 1:**
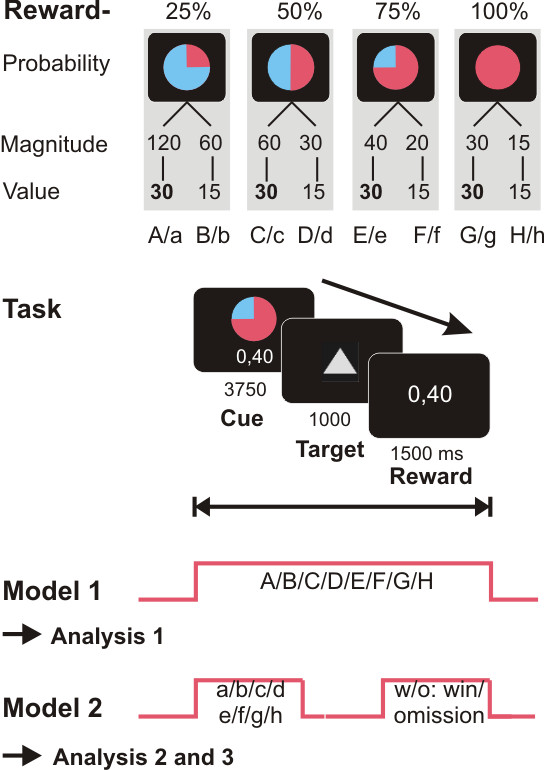
**Reward task**. Cues representing probabilities (25, 50, 75, and 100 percent) and course of the delayed incentive task as used during training and scanning: Subjects first saw the cue and were instructed to expect a certain amount of money (magnitude) at the announced probability during the delay. To preserve themselves the chance to win, they had to react correctly to the target with a button press. Reward was displayed dependent on the previously announced probabilities. For Model 1, regressors were defined over the whole length of the trial, for Model 2, Cue (expectation phase) and Reward (outcome phase) were modeled separately.

**Figure 2:**
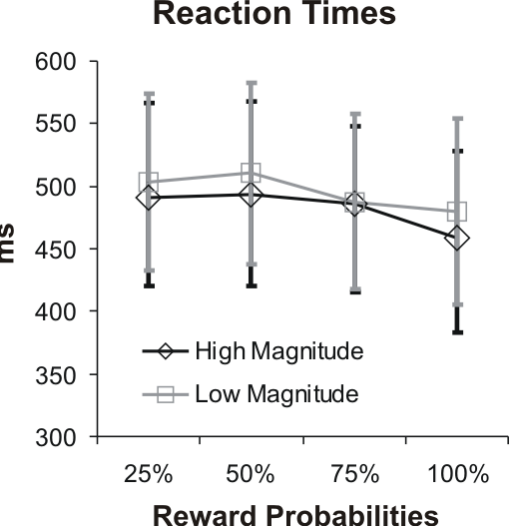
**Reaction times**. Mean reaction times during scanning of the reward task were significantly (p < 0.05) faster in the trials with high reward as compared to the trials with low reward value level. Within each level of expected reward value, significant accelerations of mean reaction times with higher probabilities were found on trials with 50% and 100% probability.

We used a monetary incentive task (figure 1) with a parametric variation of probabilities (25%, 50%, 75% and 100%) to win an announced amount of money (120, 60, 40, 30, 20 or 15 Eurocent). The different amounts of money (magnitude of reward) were assigned to the different reward probabilities in order to create eight different trial types of different probabilities but with two stable levels of expected reward values (2 value levels: 30 and 15 Eurocent, figure 1; expected reward value = probability × magnitude). This allowed analyzing reward value and uncertainty independently while probability and magnitude were defined as mutually dependant variables. Each session consisted of 96 trials (6250 ms each; 12 trials for each of the eight types: 25%-120¢, 25%-60¢, 50%-60¢, 50%-30¢, 75%-40¢, 75%-20¢, 100%-30¢, 100%-15¢). Each trial started with one of eight different indications (cue, 3750 ms) of the probability and the amount of money to win later on in this specific trial. After this expectation phase, subjects had to correctly react with a left or right button press (index or middle finger of their right hand) on two symbols, a square or a triangle (target) within a fixed interval of 1s. Subjects were notified in advance about the symbol-to-button press relation (square/right button, triangle/left button or vice versa). In reacting correctly they preserved themselves the previously announced chance to win the announced amount of money. Depending on the reward probabilities, subjects were not rewarded despite pressing the correct button in a number of trials (omission trials). Incorrect button presses resulted in a feedback of zero Eurocents. Win and omission trials as well as the eight trial types appeared in a random order. To ensure that all trials included a button press of any kind, subjects were told that they would lose 1 Euro if no button press occurred. Feedback (outcome, 1500 ms) followed the targets disappearance and notified the subjects of the amount of money (120, 60, 40, 30, 20, 15 or 0 Eurocent) they won in the trial. Reaction times (see figure 2) and errors were registered. Right before scanning, all subjects completed 2 sessions (60 trials/10 min each) of a practice version of the task. Contingencies between symbols and probabilities were explained beforehand. Subjects were told in advance that they could not win real money in the practice trial. All subjects had a performance of more than 95% correct trials during practice and could easily name the significance of each of the symbols used afterwards. In the scanner, during acquisition of the functional images, participants performed two sessions of the task (96 trials, 16 min each). The same stimuli as in the practice version were used but in a different, randomized order. The fact that we did not counterbalance the two colors (blue and red) in the task may represent a possible but very unlikely source of confounds.

### fMRI acquisition

A 3.0 Tesla Siemens ALLEGRA Scanner (Siemens, Erlangen, Germany) equipped with a head coil was used to acquire T1 anatomical volume images (1 × 1 × 1 mm voxels) and functional MR images. 23 sagittal slices were acquired with an image size of 64 × 64 pixels and a FoV of 192 mm. Slice thickness was 3 mm with a 0.75 mm gap resulting in a voxel size of 3 × 3 × 3.75 mm. Images were centered on basal structures of the brain including subcortical regions of interest (basal ganglia and prefrontal regions). Functional images were recorded using a T2*-sensitive gradient echo planar sequence measuring changes in BOLD-contrast. 650 volumes were obtained during each of the two sessions at a TR of 1500 ms (TE 35 ms, flip angle 90°).

### fMRI analysis

Image processing and statistical analysis were carried out using Statistical Parametric Mapping (SPM5, Friston, The Wellcome Department of Cognitive Neurology, London, UK). Preprocessing of the functional scans included realignment to correct for motion artifacts, slice timing, spatial normalization to a standard template (Montreal Neurological Institute, MNI) and smoothing with a 6 mm Gaussian kernel. Intrinsic autocorrelations were accounted for by AR(1) and low frequency drifts were removed via high pass filter with a cutoff frequency of 1/128 Hz.

**Figure 3:**
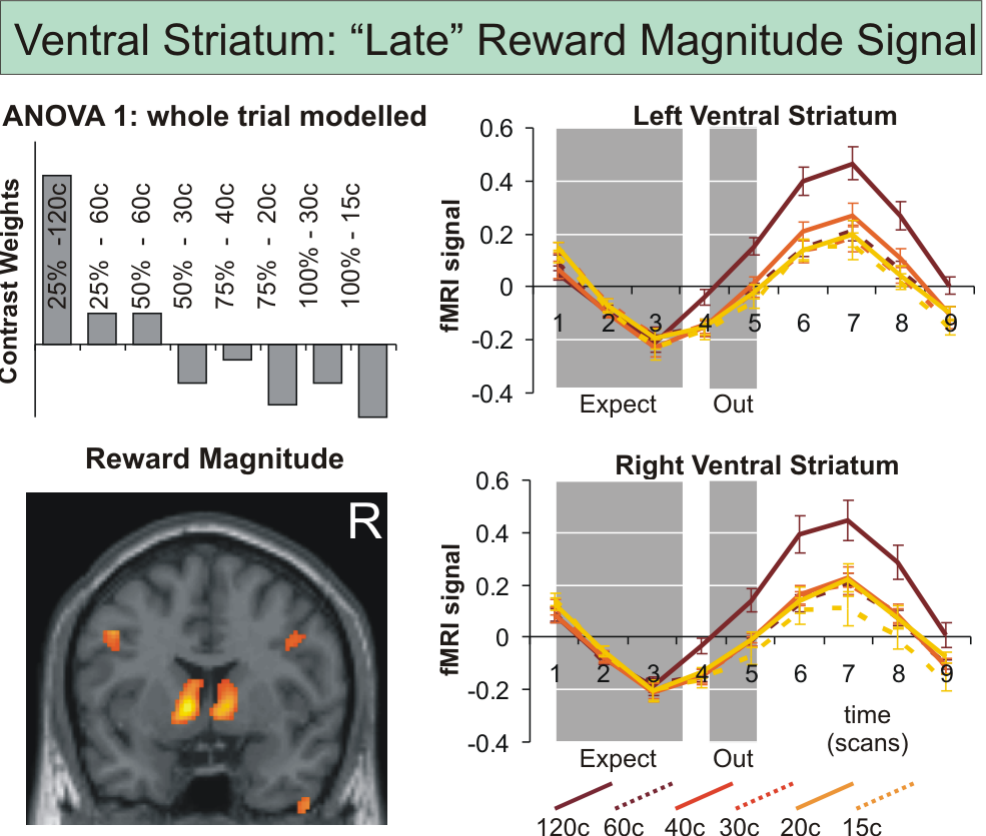
**fMRI results related to expected reward magnitudes: ventral striatum**. Significant (p < 0.05 FDR corrected) bilateral ventral striatal fMRI involvement as revealed by the repeated measures ANOVA 1 over the whole trial with eight conditions (A: 25%-120¢, B: 25%-60¢, C: 50%-60¢, D: 50%-30¢, E: 75%-40¢, F: 75%-20¢, G: 100%-30¢, H: 100%-15¢) set up to model effects of different reward magnitude. On the right panel, time courses (1^st ^eigenvariate of the fMRI signal intensity as provided by standard SPM functions and standard errors) in significant voxels of left and right ventral striatum are demonstrated. Mean-corrected fMRI signal time courses were extracted for each subject and were averaged event-related to depict the fMRI signal. Grey shades indicate the period when reward expectation took place relative to the delayed MR signal. The fMRI signal peaks around scan 7 after onset of the trial (scan 1) with significantly (p < 0.005) higher values for scan 7 than for scan 3 in all eight conditions.

**Figure 4:**
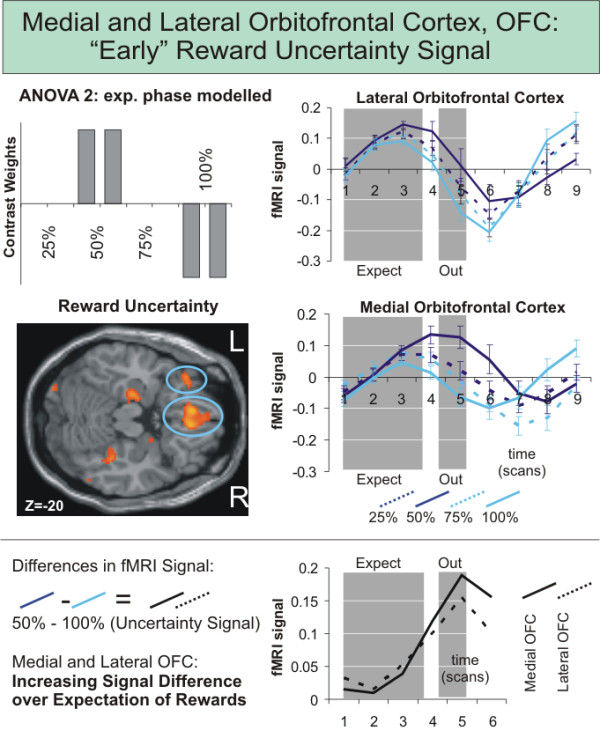
**fMRI results related to expected reward uncertainty: orbitofrontal cortex**. Significant (p < 0.05 FDR corrected) medial and left lateral orbitofrontal involvment as revealed by the repeated measures ANOVA 2 over the expectation phase with eight conditions (a: 25%-120¢, b: 25%-60¢, c: 50%-60¢, d: 50%-30¢, e: 75%-40¢, f: 75%-20¢, g: 100%-30¢, h: 100%-15¢) set up to model effects of reward uncertainty. Signal time courses in both regions are depicted in the right panel. Mean-corrected fMRI signal time courses (1^st ^eigenvariate of the fMRI signal intensity as provided by standard SPM functions and standard errors) were extracted from the two significant orbitofrontal ROIs for each subject and were averaged event-related to depict the fMRI signal. Grey shades indicate the period when reward expectation took place relative to the delayed MR signal. The fMRI signal peaks around scan 3 after onset of the trial (scan 1) with significantly (p < 0.005) higher values for scan 3 than for scan 7 in all eight conditions. Differences in signal time courses (50%, highest uncertainty and 100%, lowest uncertainty) increased from scan 1 to 5 over the course of reward expectation.

After preprocessing, first level analyses were performed on each subject estimating the variance of each and every voxel according to the General Linear Model. Regressors (see figures 1, 3 and 4) for each of the eight trial types were defined in two different ways to capture early and late BOLD responses: For Model 1 each regressor modeled one of the eight trial types (A: 25%-120¢, B: 25%-60¢, C: 50%-60¢, D: 50%-30¢, E: 75%-40¢, F: 75%-20¢, G: 100%-30¢, H: 100%-15¢) spanning the entire time interval of one trial, from presentation of the cue to the outcome phase. In this, we stayed close to the models used by Hsu et al. [4] and Tobler et al. [6] who did not model cues, responses and outcomes separately. Model 2 had again the eight regressors to model the expectation phases (cue) of each trial (a: 25%-120¢, b: 25%-60¢, c: 50%-60¢, d: 50%-30¢, e: 75%-40¢, f: 75%-20¢, g: 100%-30¢, h: 100%-15¢), another 14 regressors to model the phase of the different outcomes depending on reward expectation (exp. 25%-100%) and actual outcome (win/omission): a-w: 25%-120¢/win, a-o: 25%-120¢/omission, b-w: 25%-60¢/win, b-o: 25%-60¢/omission, and so on. An additional regressor was defined to model the button press. Phases were each modeled as a boxcar function and convolved with the hemodynamic response function. The six realignment parameters modeling residual motion were also included as regressors in each of the two models.

The contrast images of parameter estimates from Model 1 and 2 were then combined in second level group analyses, treating intersubject variability as a random effect to account for interindividual variance. We computed three separate ANOVAs. The first ANOVA (ANOVA 1) comprised eight conditions according to the regressors of Model 1 (entire trial duration). The second ANOVA (ANOVA 2) had as conditions the eight expectation regressors from Model 2. Finally, the third ANOVA (ANOVA 3) comprised the fourteen outcome conditions formulated in Model 2. Within each ANOVA, conditions were weighted with contrasts to model effects of reward magnitude (figure 3), probability, uncertainty (figure 4), expected value (regressors A/a, C/c, E/e > regressor B/b, D/d, F/f) and prediction error (difference between reward expected and actually received) according to our hypotheses. For statistical maps we used conservative thresholds of p < 0.05 FDR corrected for multiple comparisons and an extent threshold at the cluster-level of p < 0.05.

For the analysis of the signal time course data to investigate the fMRI signal independently of any model, functional regions of interest (ROIs) of group activations were defined at p < 0.05 to p < 0.001 FDR corrected. Even more conservative thresholds were chosen in the case of large clusters like the medial orbitofrontal cortex to only include the most significant voxels. For each subject, the first eigenvariate of signal intensities of all voxels within a predefined functional region was extracted to obtain fMRI signal time series. The event-related time courses as depicted in figure 3 and 4 were obtained by first extracting series of 9 time points (TRs) starting with the onset of each trial, and then averaging over all trials of the same type, the two runs and subjects. T-tests were used to compute differential effects in the voxel time series using external software (Microsoft Excel, Statsoft Statistica).

## Results

### Behavioral responding

Subjects pressed the correct button within the required time in 98% of the trials. A multivariate analysis of variance for repeated measures on mean reaction times revealed a significant main effect for high and low expected reward values (F1,14) = 14.77, p = 0.002), and levels of probability (F(3,12) = 17.66, p < 0.001). Due to the similar trends of mean reaction times for high and low reward values over increasing probabilities (see figure 2), interaction of both factors was not significant (F(3,12) = 1.43, p = 0.283). Single contrasts (paired t-tests) within each level of expected reward value, yielded significant differences of mean reaction times on trials with 50% and 100% probability (high level: t(14) = 4.68, p < 0.001; low level: t(14) = 4.64, p < 0.001). Comparisons between levels of expected reward value at different probabilities revealed significantly faster reaction times for trials with higher levels than for lower levels at probabilities of 50% (t(14) = -2.36, p = 0.033) and 100% (t(14) = -2.94, p = 0.011). For probabilities of 25% (t(14) = -1.81, p = 0.091) and 75% (t(14) = -0.16, p = 0.873) mean reaction times did not differ (see figure 2).

### fMRI Results

#### ANOVA 1, modeling effects over the whole trial

When testing on effects of magnitude (figure 3) significant results were obtained in bilateral ventral striatum and anterior insula/inferior frontal gyrus (table 1). This result pattern was the same as that obtained by contrasting high versus low reward value levels irrespective of reward uncertainty. The contrasts modeling increasing reward probability and uncertainty did not reveal activation of the primary reward network.

**Table 1:** fMRI Results: ANOVA 1, modeling effects over the whole trial

Analyses performed/Cerebral region	R/L	NV	t-value	Coordinates of peak activity		
				x	y	z
**ANOVA 1: Effects over whole trial**						

***ANOVA: increasing reward magnitude, t = 2.68***						
ventral striatum	R	441	4.38	10	6	2
	L	267	5.31*	-8	8	2
anterior insula/IFG	R	1101	7.67*	32	26	-8
	L	1083	7.12*	-40	20	-12
orbitofrontal cortex	L	255	4.21	-30	54	-4
dorsal anterior cingulate cortex		555	4.64	-10	26	32
		135	4.43	10	28	34
medial temporal gyrus	R	441	4.67	54	-38	-6
	L	106	4.50	-58	-36	-16
DLPFC	R	403	4.65	44	18	24
		159	3.72	40	36	16
	L	215	3.92	-46	6	38
lateral occipital cortex	R	2160	7.45*	42	-90	-6
	L	2767	6.41*	-40	-90	-8

***ANOVA: high > low reward value level, t = 4.49***						
ventral striatum	L	14	5.17*	-6	10	2
anterior insula/IFG	R	13	5.00	40	26	-14

***ANOVA: increasing reward probability***						
n.s.						

***ANOVA: increasing reward uncertainty, t = 3.37***						
lateral occipital cortex	R	612	9.17*	36	-96	-4
	L	201	5.82*	-36	-92	-4
DLPFC	L	49	4.57	-52	10	22
	L	33	4.56	-54	-2	32
hippocampal area	L	41	4.28	-18	-12	-16

Group maximum t-values and MNI coordinates of activation foci for the three repeated measures ANOVAs computed on the second level. Statistical maps were generally thresholded at p < 0.05 FDR corrected for multiple comparisons and FWE corrected where indicated. Results were extent threshold corrected at 0.05 at the cluster level.

*: FWE corrected; #: part of the cluster above

#### ANOVA 2, modeling effects over expectation phase

**Figure 5:**
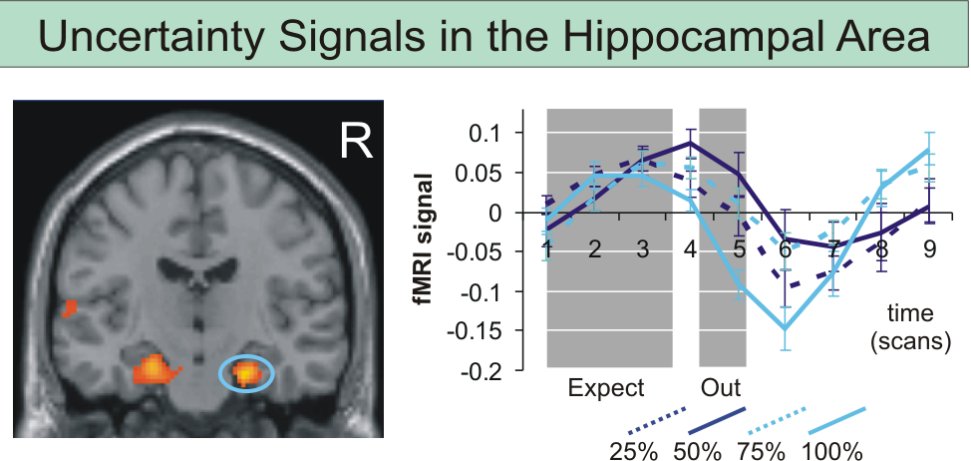
**fMRI results related to expected reward uncertainty: hippocampus**. Significant (p < 0.05 FDR corrected) involvement of the bilateral hippocampal area as revealed by the repeated measures ANOVA 2 over the expectation phase with eight conditions (a: 25%-120¢, b: 25%-60¢, c: 50%-60¢, d: 50%-30¢, e: 75%-40¢, f: 75%-20¢, g: 100%-30¢, h: 100%-15¢) set up to model effects of reward uncertainty. Signal time courses (1^st ^eigenvariate of the fMRI signal intensity as provided by standard SPM functions and standard errors) in the significant right hippocampal area are depicted in the right panel.

To analyze effects during the expectation period together with responses early during the trial, an ANOVA of main effects of the eight different expectation regressors was computed. We again tested on significant effects for contrasts modeling increasing reward magnitude, value, probability and uncertainty (table 2). Left-sided ventral striatal involvement was again found for the contrast modeling reward magnitude; contrasts modeling reward value and probability yielded no significant results. The contrast modeling reward uncertainty now revealed significant involvement of medial prefrontal and left orbitofrontal cortex (figure 4), and bilateral hippocampal area (figure 5).

**Table 2:** fMRI results: ANOVA 2, modeling effects over expectation phase:

Analyses performed/Cerebral region	R/L	NV	t-value	Coordinates of peak activity		
				x	y	z
**ANOVA 2: Effects over expectation phase**						

***ANOVA: increasing reward magnitude t = 3.47***						
ventral striatum	L	34	4.39	-12	6	0
gyrus fusiformis	L	90	5.14	-30	-64	-10
		75	4.54	-24	-50	-12
	R	121	4.42	34	-60	-14
lateral occipital cortex	R	435	5.94*	42	-90	-4
	L	215	5.45*	-20	-98	-10

***ANOVA: high > low reward value level***						
n.s.						

***ANOVA: increasing reward probability***						
n.s.						

***ANOVA: increasing reward uncertainty, t = 2.94***						
MPFC		1184	5.53*	2	50	-12
			5.48*	-8	34	-22
lateral orbitofrontal cortex	L	87	4.04	-36	34	-18
DLPFC	L	76	4.08	-26	26	44
hippocampal area	L	310	5.09	-22	-18	-22
	R	189	5.43*	22	-16	-24
amygdala	R	#	3.48	22	0	-20
						
medial temporal gyrus	L	132	4.80	-60	-30	8
precuneus	R	1177	6.06*	14	-54	12
						
lateral occipital cortex	R	1265	9.19*	34	-96	-4
	L	297	5.68*	-34	-94	-4

Group maximum t-values and MNI coordinates of activation foci for the three repeated measures ANOVAs computed on the second level. Statistical maps were generally thresholded at p < 0.05 FDR corrected for multiple comparisons and FWE corrected where indicated. Results were extent threshold corrected at 0.05 at the cluster level.

*: FWE corrected; #: part of the cluster above

#### ANOVA 3, modeling effects over outcome phase

Contrasting conditions with positive prediction error (win) versus omissions modeling a negative prediction error revealed an effect in bilateral ventral striatum and medial prefrontal cortex (table 3).

**Table 3:** fMRI results: ANOVA 3, modeling effects over outcome phase

Analyses performed/Cerebral region	R/L	NV	t-value	Coordinates of peak activity		
				x	y	z
**ANOVA 3: Effects over outcome phase**						

***ANOVA modelling prediction error t = 2.69***						
ventral striatum	L	3794	6.56*	-10	10	-6
	R	#	5.51*	10	12	-2
medial prefrontal cortex		2863	5.62*	-8	36	-12
lateral occipital cortex	R	1575	7.98*	32	-96	-2
	L	700	7.06*	-32	-98	-2

Group maximum t-values and MNI coordinates of activation foci for the three repeated measures ANOVAs computed on the second level. Statistical maps were generally thresholded at p < 0.05 FDR corrected for multiple comparisons and FWE corrected where indicated. Results were extent threshold corrected at 0.05 at the cluster level.

*: FWE corrected; #: part of the cluster above

#### Analysis of time courses

The detailed analysis of voxel time courses from the main dopaminergic brain areas involved (ventral striatum, medial and lateral orbitofrontal cortex) revealed different temporal dynamics of the brain regions involved in the signaling of reward magnitude/value and those involved in the signaling of reward uncertainty. While the hemodynamic response in the ventral striatum showed a peak around scan 7 after onset of the trial (scan 1) for all eight expectation conditions, peak activations were much earlier, around scan 3 to 4, in orbitofrontal regions. Paired t-tests on the individual signal time course data confirmed, that ventral striatal activation in all eight conditions was significantly higher for scan 7 than for scan 3 or 4 (p < 0.005 for all eight conditions). In medial and lateral orbitofrontal cortex activation for scan 3 was significantly higher than for scan 7 (p < 0.05 for all eight conditions). Signal differences between highest (50%) and lowest (100%) uncertainty showed a linear increase from scan 1 to 5 (figure 4).

## Discussion

Here we studied whether fMRI activation and its signal characteristics in different dopaminergic brain areas can be described as a function of reward uncertainty and value or the components of reward value, magnitude and probability, as shown for average firing rates of dopamine neurons in animal experiments. We suggested that differences in BOLD signal characteristics may be observed, parallel to the differences in firing rate characteristics for reward value and uncertainty. Subjects were asked to expect monetary rewards of different magnitudes, probabilities and uncertainty and were confronted with receipt or omission of these. We constructed trials with one high and one low reward value level (value = magnitude × probability) and with no (0% and 100%), intermediate (25% and 75%) and high (50%) uncertainty. We found that the coding of expected reward value and particularly reward magnitude as one of its components [13] is related to activation in the ventral striatum with a BOLD signal peak relatively late after trial onset. Activation during this late phase of the trial was significantly higher when compared to the early phase closer to the onset of the trial. As demonstrated in earlier studies [9,10], reaction times as a measure of motivation paralleled the quantity of fMRI activation to an increased reward value, with faster reactions at higher reward magnitudes and probabilities.

In contrast to the late BOLD signal peak in the ventral striatum, the peak of the lateral and medial orbitofrontal BOLD signal occurred very close to the onset of the trial. Thus, we were able to confirm the suggestion by Hsu and colleagues [4] that the different dopaminergic pathways involved may be reflected in different signaling time courses in different brain regions. Present results therefore parallel the finding of differential phasic and tonic signals of dopaminergic cells for reward value and uncertainty as demonstrated in monkeys [3]. Though these parallels are intriguing it is of note that we do not claim that different signaling time courses do directly reflect differential phasic and tonic signals of dopaminergic cells in experimental animals.

Besides activation of the insula [14], the medial prefrontal cortex [15] and the prefrontal cortex [16] previous imaging studies [4,6] reported activation in the lateral orbitofrontal cortex related to the processing of reward uncertainty. However, neither variations of that signal over a range of uncertainty values [4], nor examinations of temporal properties of the BOLD signal [6] became evident from these studies. Our results integrate these prior findings on orbitofrontal functioning: we replicate that reward uncertainty is related to activation in the lateral orbitofrontal cortex, and extend previous findings by demonstrating that the corresponding signal peaks comparably close to trial onset.

Concerning ventral striatal activation, the signal might have not exclusively been related to the expectation phase but could have also been elicited by the rewards obtained during the outcome phase. However, when modeling the prediction error (difference between reward expected and actually received) during the outcome phase in ANOVA3, ventral striatal activation was related only to a contrast of win versus omission trials with equal monetary rewards but not to the amounts of money won. Furthermore, ventral striatal activation was related to expected reward value and its components of expected reward magnitude and probability in a number of previous studies [6,9,10,13,17].

Of great interest and open to discussion is the question whether or how tonic, longer-lasting dopamine responses mediating uncertainty signals are indeed related to early BOLD responses and whether or how short and phasic dopamine responses mediating reward value are related to late BOLD responses. There is evidence for a direct relation between the quantity (% BOLD signal change) of an fMRI signal and average firing rates of neurons [18]. It is also well established that projections from midbrain neurons mediate reward related dopamine release in the ventral striatum and prefrontal cortex. However, knowledge about the relation between instantaneous neuronal firing rates and the time course of the BOLD signal is scarce. Also, the BOLD signal is not a direct measure of neuronal activation, but appears to be the result of interactions of neurotransmitters with presynaptic and postsynaptic receptors. For example, it has previously been shown that the BOLD signal in the ventral striatum is mediated by postsynaptic D1 receptors [19] with receptor-related indirect effects on the BOLD signal characteristics [20]. We therefore suggest that the temporal differences in the signals as seen in our study may not reflect different processing speeds and may not point to preceding or succeeding events but are due to such indirect effects. Rather, early and late BOLD responses may point to a processing of reward value and uncertainty via two different pathways thereby paralleling the findings from monkey neurons of tonic and phasic firing rates. However, a potential direct connection or link between firing rates and BOLD signal remains unclear.

Beyond differentiating the two signaling pathways, we succeeded in replicating a notable characteristic of the tonic signal as measured in monkeys showing a gradual increase of the difference in signal between trials with no uncertainty and with highest uncertainty over time (figure 4). Demonstrating that the BOLD signal in the orbitofrontal cortex upon expectation of uncertain rewards shows parallels with firing rates of dopaminergic neurons, supports the notion that our measures of BOLD contrasts are in good agreement with previous animal research on the neuronal level [3]. This observed gradual increase could represent the gradual increase of perceived uncertainty over the course of reward expectation, with the highest signal just at the point before it is resolved. In terms of learning, items indicating uncertain outcomes provide indeed information [3]: While with cues indicating low degree of uncertainty (a reward at 100%) no new information is anticipated, cues indicating a high degree of uncertainty (a reward at 50%) suggest that something unexpected will happen and that something new can be learned. In order to trigger learning, such a signal should have its maximum just at the point in time when the uncertainty is resolved and information provided, as shown for the differential uncertainty signals in monkey neurons and the differential activation in our study (figure 4).

In this context, bilateral activation of the hippocampal area in the contrast of trials with and without uncertainty may be interpreted in a sense that reward uncertainty indeed activates networks related to uncertainty in more general contexts. Strange et al. [21] showed that left anterior hippocampal activation is related to the entropy of event streams, with entropy defined as a measure of the expected uncertainty of events in a given context. Extending earlier findings that linked hippocampal activation to novelty detection [22], Strange et al. showed that hippocampal activation is related to the probabilistic structure of observed events. Like activation in orbitofrontal regions, activation of the bilateral hippocampal area in response to reward uncertainty in our study showed an earlier peak as compared to activation related to reward value. Interactions between dopamine networks and hippocampal activation have been described in the context of psychiatric disorders [23,24]. Particularly the tonic dopamine response linked to reward uncertainty by Fiorillo et al [3] is modulated by limbic inputs from hippocampus and basolateral amygdala [25,26]. Furthermore, genetic influences on the D2 receptor which has been suggested to be particularly sensitive to the tonic responses [7,8], have been shown to modulate interactions between the hippocampus and other brain areas in a probabilistic learning task [27]. We take this as further support for our notion that early fMRI responses may parallel the role of tonic dopamine signaling when processing reward uncertainty.

## Conclusion

Replicating findings from animal research [3], we demonstrate that dopaminergic pathways coding reward value and uncertainty are not only represented by the involvement of differential brain regions but can be differentiated by distinct signaling properties. While reward uncertainty related to tonic dopaminergic signals in monkeys results in relatively early BOLD responses in lateral orbitofrontal regions, expected reward value related to phasic signals is coded independently in the ventral striatum by a relatively later BOLD response. The finding of distinct BOLD signal characteristics parallels findings from distinct dopaminergic signaling pathways in animal research.

## Authors' contributions

BA participated in the conception and design of the study and data acquisition, performed analysis and interpretation of the data and drafted the manuscript. BH carried out the acquisition of the data, participated in the analysis and interpretation of the data and revised the manuscript. GG was involved in the conception and design of the study, analysis and interpretation of the data and revising the manuscript. MS participated in the conception and design of the study, interpretation of the data and revising the manuscript. All four authors read and approved the final manuscript.
